# Molecular and immune landscape of melanoma: a risk stratification model for precision oncology

**DOI:** 10.1007/s12672-025-02497-0

**Published:** 2025-05-04

**Authors:** Miao Sun, Junliang Wu

**Affiliations:** 1 Operating Room, Department of Anesthesiology, West China Hospital, Sichuan University/West China School of Nursing, Sichuan University, Chengdu, Sichuan China; 2 Department of Plastic and Burn Surgery , West China School of Medicine, West China Hospital, Sichuan University, Chengdu, 610041 China

**Keywords:** Melanoma stratification, Immune infiltration, Prognostic biomarkers, Gene expression profiling, TCGA data analysis

## Abstract

**Background:**

Melanoma is a highly aggressive skin cancer with significant heterogeneity in immune infiltration and clinical outcomes. Accurate risk stratification is essential for improving personalized treatment strategies.

**Methods:**

This study utilized data from The Cancer Genome Atlas (TCGA) to explore immune-related gene expression in melanoma. Single-sample gene set enrichment analysis (ssGSEA) was employed to classify patients into high and low immune groups. Tumor microenvironment (TME) characteristics, including immune cell infiltration, HLA gene expression, and TME scores, were analyzed. Prognostic genes were identified using univariate and multivariate Cox regression analyses. A risk score model and nomogram were constructed, and gene set enrichment analysis (GSEA) and gene set variation analysis (GSVA) were conducted to explore relevant signaling pathways.

**Results:**

ssGSEA-based classification revealed significant differences between high and low immune groups in terms of immune infiltration and HLA gene expression. The risk model incorporated immune-related genes such as GBP2, SEMA4D, and KIR2DL4, which demonstrated distinct tumor expression profiles and strong prognostic value. GSEA and GSVA analyses uncovered critical immune-related and oncogenic pathways linked to risk stratification. A predictive nomogram integrating molecular risk scores and clinical variables improved prognostic accuracy. Computational immune deconvolution highlighted CD8+ T cell infiltration as a key prognostic factor. To validate the functional role of candidate genes, *KIR2DL4* was silenced in A375 melanoma cells using shRNA. Knockdown efficiency was confirmed by qRT-PCR. Functional assays revealed that *KIR2DL4 silencing significantly reduced cell proliferation*, as assessed by MTT assay, and *impaired migratory capacity*, as demonstrated by wound healing assay. These in vitro findings support the computational predictions and suggest that KIR2DL4 may play a tumor-promoting role in melanoma.

**Conclusion:**

This study provides a robust immune-related prognostic model for melanoma. It underscores the value of immune gene expression and T cell infiltration, particularly CD8+ T cells, in predicting patient outcomes. The model facilitates personalized treatment decisions and advances precision oncology approaches in melanoma. The integration of transcriptomic analysis with experimental validation confirms the tumor-promoting role of KIR2DL4 and enhances the translational value of the model in guiding precision immunotherapy.

## Introduction

Malignant melanoma, a severe form of skin cancer, originates from melanocytes, the pigment-producing cells [1]. It is known for its rapid progression, metastasis, and poor prognosis if not detected early [2]. Though it constitutes a small fraction of skin cancer cases, it is responsible for the majority of skin cancer deaths. Key risk factors include significant ultraviolet radiation exposure, a family history of melanoma, and genetic predispositions [3]. Clinically, malignant melanoma is marked by the uncontrolled proliferation of melanocytes, often beginning as an atypical mole or skin lesion [4]. The global incidence of malignant melanoma is on the rise, making it a major public health concern. Early detection and surgical excision are essential for treating localized melanoma [5]. In advanced stages, treatment may require a combination of surgery, immunotherapy, radiation, and chemotherapy [6]. Recent advancements in targeted therapies and immune checkpoint inhibitors have transformed the treatment landscape, providing new hope for improved survival rates [7].

Immunotherapy has revolutionized the treatment of malignant melanoma, an aggressive cancer characterized by rapid growth and spread [8]. This therapeutic approach leverages the body’s immune system to recognize and fight cancer cells [9]. The effectiveness of immunotherapy in melanoma can be attributed to a deeper understanding of the tumor microenvironment and the intricate interactions among various immune cells, including T cells, B cells, dendritic cells, and macrophages [10]. Cytotoxic T lymphocytes are particularly crucial in the anti-tumor immune response, as they can directly kill melanoma cells when activated [11]. However, melanoma cells often evade immune detection by manipulating immune checkpoints, leading to T cell inactivation [12]. Checkpoint inhibitors, such as CTLA-4 and PD-1/PD-L1 inhibitors, have been developed to block these pathways, reactivating T cells to target the tumor [13]. Dendritic cells, as antigen-presenting cells, are also central to melanoma immunotherapy [14]. Enhancing their capacity to present tumor antigens effectively is a strategy to stimulate a strong anti-melanoma response. Researchers are also investigating the roles of other immune cells, like B cells and macrophages, in the melanoma microenvironment [15]. Understanding their interactions with melanoma cells and other immune components could reveal new therapeutic targets and strategies.

In recent years, bioinformatics has become an essential tool in understanding and combating complex diseases like malignant melanoma. This aggressive skin cancer poses a significant challenge due to its ability to evade and suppress the immune system. While immunotherapy has opened new treatment avenues, offering hope for improved survival rates, the underlying biological mechanisms of its successes and failures are complex and not fully understood. Bioinformatics provides powerful tools to dissect these complexities. Our article explores how bioinformatics analysis can unravel the intricate relationship between immunotherapy and malignant melanoma. By integrating large datasets from genomic, proteomic, and transcriptomic studies, we aim to identify patterns and molecular signatures that influence the response to immunotherapy. This includes discovering biomarkers to predict treatment response, understanding the genetic basis of the immune evasion strategies used by melanoma cells, and exploring the tumor microenvironment’s role in shaping the immune response.

## Method

### Data acquisition

Our study utilized publicly available datasets from The Cancer Genome Atlas (TCGA) database, specifically focusing on genomic and clinical data from patients diagnosed with malignant melanoma. This data encompassed gene expression profiles, DNA methylation, copy number variation, mutation information, and patient survival data. Prior to analysis, we preprocessed the data to ensure quality and consistency, which included normalizing gene expression data, annotating genomic coordinates, and imputing missing values where necessary. We also filtered out low-quality or incomplete samples to maintain the integrity of the analysis.

### Differential expression analysis

We began by acquiring RNA sequencing and clinical data for malignant melanoma from TCGA. After quality control and normalization procedures, we employed the DESeq2 package to conduct differential expression analysis between tumor and normal samples, focusing on genes with significant expression changes. Multiple hypothesis testing was adjusted using the Benjamini–Hochberg method to control the false discovery rate.

### GO and KEGG enrichment analysis

To determine the biological significance of differentially expressed genes in malignant melanoma, we conducted gene ontology (GO) and Kyoto Encyclopedia of Genes and Genomes (KEGG) pathway enrichment analyses. Following differential expression analysis, we ranked genes based on their significance and selected the top differentially expressed genes for further investigation. For GO enrichment, we used the BiNGO plugin in Cytoscape or the clusterProfiler package in R to map all differentially expressed genes to GO terms in the categories of biological processes, cellular components, and molecular functions. Similarly, for KEGG pathway enrichment analysis, we used the clusterProfiler package to identify critical pathways significantly enriched among the differentially expressed genes.

### GSEA and GSVA enrichment analysis

To gain a comprehensive understanding of the molecular changes associated with malignant melanoma, we employed gene set enrichment analysis (GSEA) and gene set variation analysis (GSVA). We used GSEA software to determine whether a predefined set of genes showed statistically significant, concordant differences between two biological states (e.g., tumor vs. normal). We selected gene sets from the Molecular Signatures Database (MSigDB), focusing on hallmark gene sets and oncogenic signatures. GSEA provided an enrichment score reflecting the degree to which a gene set was overrepresented at the extremes of the entire ranked list of genes. Statistical significance of the enrichment scores was determined through permutation testing. GSVA was performed using the GSVA package in R, a non-parametric, unsupervised method that estimates variation of gene set enrichment through the samples in an expression dataset. Unlike GSEA, GSVA provided scores for each sample, converting gene expression data into a pathway-level expression matrix, which is useful for understanding the variation of pathway activity over a sample population and identifying potential biomarkers or therapeutic targets.

### Construction of the prognostic prediction model

We initiated our prognostic model construction by performing a univariate Cox proportional hazards regression analysis on all candidate predictors obtained from our previous differential expression and enrichment analyses. Variables with *p*-values less than 0.05 were considered significant and selected for further analysis. To refine our model and address potential overfitting due to high-dimensional data, we employed the Least Absolute Shrinkage and Selection Operator (LASSO) regression method using the ‘glmnet’ package in R. The optimal penalty parameter lambda was determined using ten-fold cross-validation. Following variable selection with LASSO, we validated and adjusted the model using multivariate Cox regression analysis, incorporating all significant variables from the LASSO regression into a Cox model to estimate the hazard ratios and their confidence intervals. The final model’s proportional hazards assumption was verified using Schoenfeld residuals.

### Immune cell infiltration analysis

We integrated results from CIBERSORT, TIMER, and xCell to provide a comprehensive view of the immune cell landscape. We used statistical and bioinformatics techniques to interpret the combined data, focusing on identifying dominant immune cell types, their correlation with clinical outcomes, and potential implications for immunotherapy response. By employing multiple algorithms for immune cell infiltration analysis, we aimed to obtain a robust and nuanced understanding of the immune microenvironment in malignant melanoma, enhancing the reliability of our findings and providing valuable insights into the potential immunological mechanisms driving melanoma progression and treatment response.

### Single-sample gene set enrichment analysis (ssGSEA)

In our study, we applied single-sample gene set enrichment analysis (ssGSEA) to quantify immune cell infiltration within malignant melanoma tissues. We commenced by procuring normalized and quality-controlled RNA sequencing data from melanoma samples. Utilizing the GSVA package in R, ssGSEA was performed to generate enrichment scores for each sample against a predefined collection of immune-related gene sets. These gene sets encompassed markers and pathways specific to various immune cell types and functions. The algorithm ranked all genes in each sample based on expression and calculated enrichment scores, reflecting the relative abundance or activity of each immune cell type or pathway. We then correlated these enrichment scores with clinical data to explore associations between immune infiltration patterns and patient outcomes.

### Cell culture

Human melanoma cell lines A375 was obtained from the American Type Culture Collection (ATCC)]. Cells were cultured in Dulbecco’s Modified Eagle Medium (DMEM) supplemented with 10% fetal bovine serum, 100 U/mL penicillin, and 100 μg/mL streptomycin at 37 °C in a humidified incubator with 5% CO_2_. Cells were routinely passaged at 70–80% confluence using 0.25% trypsin–EDTA and maintained in logarithmic growth phase for subsequent experiments. All cell lines were regularly tested for mycoplasma contamination and used within 10 passages after thawing.

### shRNA construct design and cloning

Short hairpin RNAs (shRNAs) were designed using the RNAi Consortium (TRC) shRNA design tool, and sequences with high predicted knockdown efficiency and minimal off-target potential were selected. Oligonucleotides encoding the shRNA sequences were synthesized, annealed, and cloned into the pLKO.1-puro vector according to the manufacturer’s instructions. A non-targeting shRNA sequence was used as a negative control. All constructions were confirmed by Sanger sequencing. Lentiviral particles were produced by co-transfecting into HEK293T cells using Lipofectamine 3000, and viral supernatants were collected 48 h post-transfection for subsequent infection of target cells.

### RNA extraction and quantitative real-time PCR (qRT-PCR)

Total RNA was extracted using the TRIzol reagent according to the manufacturer’s instructions. RNA concentration and purity were measured using a NanoDrop 2000 spectrophotometer (Thermo Scientific), and RNA integrity was assessed by agarose gel electrophoresis. One microgram of total RNA was reverse transcribed into cDNA using the PrimeScript RT reagent kit following the manufacturer’s protocol. Quantitative real-time PCR (qRT-PCR) was performed using SYBR Green. Each reaction was carried out in triplicate. Gene expression levels were normalized to GAPDH, and relative expression was calculated using the 2^−ΔΔCt^ method.

### CCK8 assay

Cell viability was evaluated using the CCK8 assay. Briefly, cells were seeded in 96-well plates at a density of 2000 cells/well and allowed to adhere overnight. After treatment with the indicated compounds or conditions for 24, 48, and 72 h, 20 μL of MTT solution was added to each well and incubated for 4 h at 37 °C. Subsequently, the supernatant was carefully removed, and 150 μL of dimethyl sulfoxide (DMSO) was added to dissolve the formazan crystals. The absorbance was measured at 450 nm using a microplate reader, and cell viability was expressed as a percentage of the control group.

### Wound healing assay

Cell migration ability was assessed using a wound healing assay. Briefly, 5 × 10^5^ cells were seeded into 6-well plates and cultured until they formed a confluent monolayer. A sterile 200 μL pipette tip was used to create a linear scratch (wound) across the cell monolayer. The detached cells were removed by washing gently with phosphate-buffered saline (PBS), and fresh serum-free medium was added. Images of the wound area were taken at 0 and 24 h after scratching using an inverted phase-contrast microscope.

## Results

### Immune profiling of melanoma reveals distinct high and low immunity subtypes with differential HLA gene expression and immune cell infiltration

**Fig. 1:**
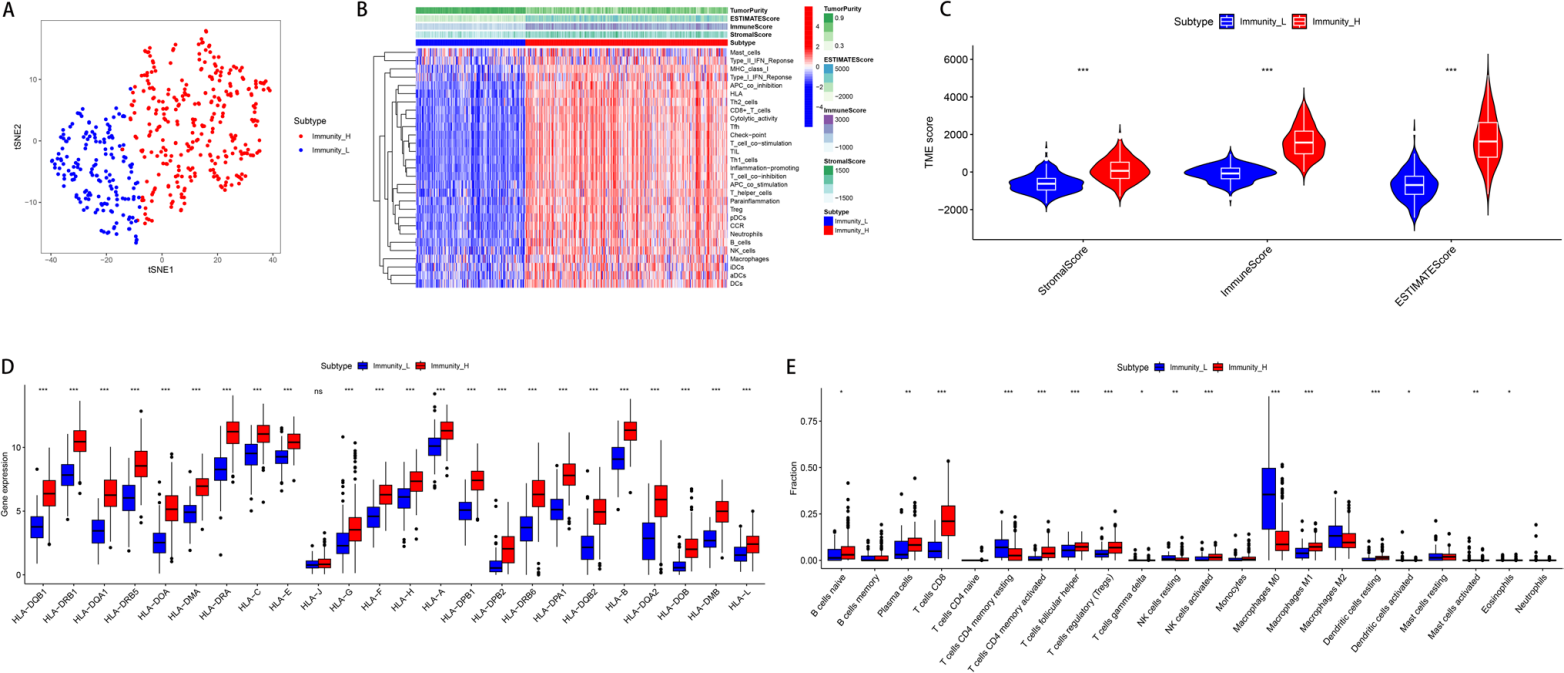
Immune profile classification and tumor microenvironment analysis in melanoma. **A** t-SNE plot demonstrating the segregation of melanoma samples into high (Immunity_H) and low (Immunity_L) immune response groups based on ssGSEA scores. **B** Heatmap showing the expression patterns of all genes and immune-related genes across the two immune groups. **C** Box plots comparing tumor microenvironment (TME) scores—including stromal score, immune score, and ESTIMATE score—between Immunity_H and Immunity_L groups. **D** Box plots showing differential expression of HLA-related genes across immune subgroups. **E** Box plots illustrating the relative abundance of various immune cell types estimated by deconvolution algorithms across the two immune groups. Statistical significance was determined using the Wilcoxon rank-sum test

In our investigation of melanoma samples from The Cancer Genome Atlas (TCGA), we employed the ssGSEA algorithm to stratify the cohort into high and low immune response groups (Fig. 1A, B). Through t-SNE analysis, we established distinct clusters corresponding to each immune profile. The high immune group (Immunity_H) demonstrated elevated Tumor Microenvironment (TME) scores, encompassing higher Immune and Stromal scores, and consequently, an increased ESTIMATEScore, indicative of a pronounced immune presence (Fig. 1C). Furthermore, we observed a marked upregulation of HLA gene expression within the Immunity_H group, suggesting a heightened antigen presentation capacity (Fig. 1D). Correlation analyses disclosed significant variations in the immune cell composition between the two groups, with the Immunity_H group exhibiting a higher fraction of critical immune cells such as activated memory CD4+ T cells and CD8+ T cells (Fig. 1E).

### Prognostic modeling in melanoma delineates risk stratification based on differential immune-related gene expression

**Fig. 2:**
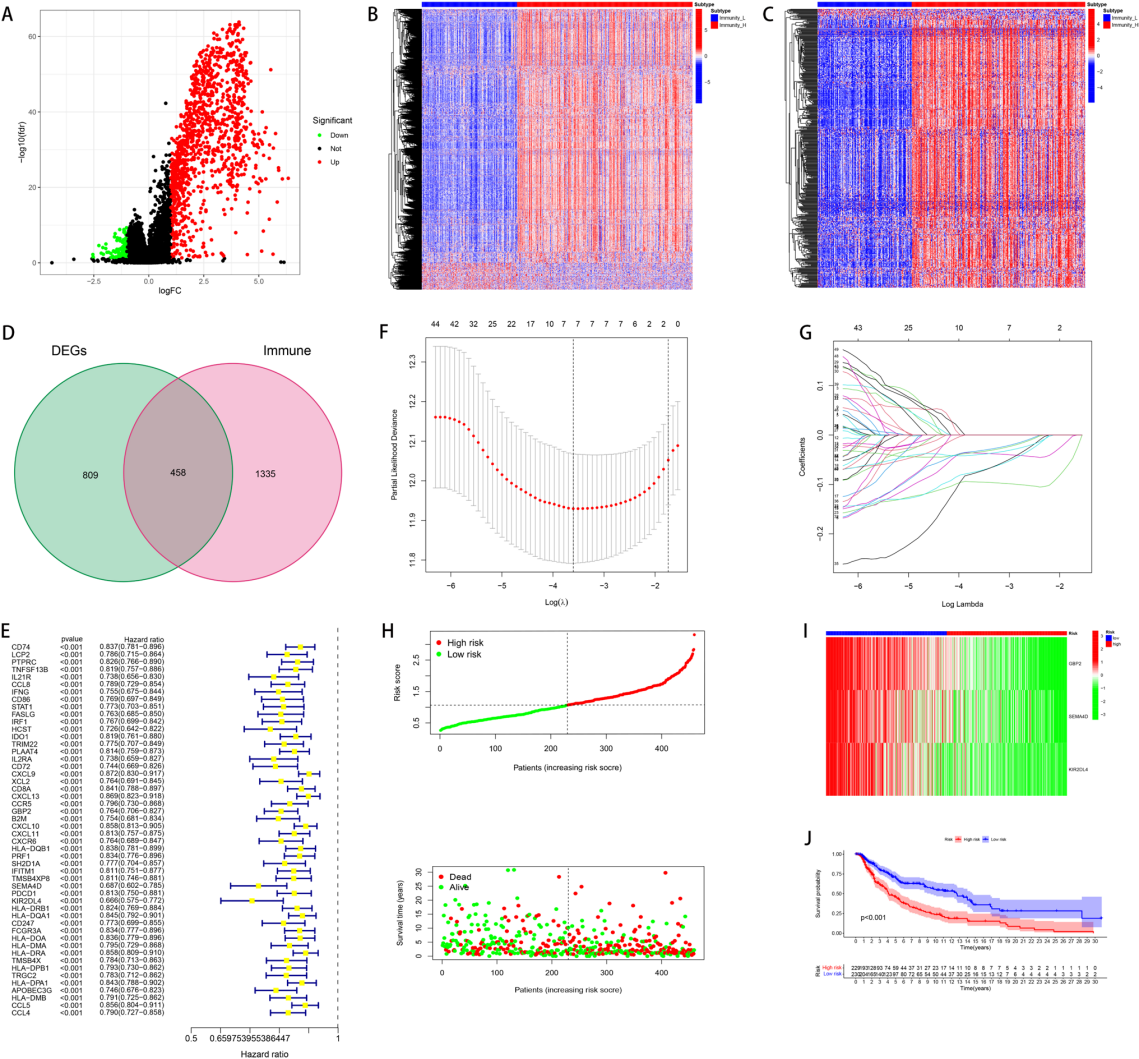
Identification of differentially expressed immune-related genes and development of a prognostic model. **A** Volcano plot displaying differentially expressed genes (DEGs) between high and low immune groups (adjusted *p*-value < 0.05, |log2FC|> 1). **B** Venn diagram showing the intersection between DEGs and immune-related gene sets. **C**–**E** LASSO regression analysis for feature selection of prognostic immune-related genes. **F** Nomogram integrating the immune-based risk score and clinical variables to predict 1-, 3-, and 5-year overall survival in melanoma patients

In our comprehensive analysis, we first performed a differential expression analysis between high and low immune response melanoma groups, which revealed a significant repertoire of differentially expressed genes (DEGs) (Fig. 2A). Heatmaps of all gene expression profiles (Fig. 2B) and those specifically of immune-related genes (Fig. 2C) exhibited distinct expression patterns between the groups, underlying the potential molecular mechanisms driving immune responses in melanoma. Through the intersection of DEGs with immune-related genes, we identified key genes for further analysis (Fig. 2D). Subsequent univariate Cox regression analysis isolated significant prognostic markers from these genes. We refined our prognostic markers using Lasso regression analysis to prevent model overfitting (Fig. 2F, G), and established a multivariate Cox regression model that delineated high-risk and low-risk patient groups based on their gene expression profiles (Fig. 2H). The survival analysis showed a clear separation in survival probability between the two risk groups (Fig. 2J), confirming the prognostic utility of our model.

### Construction and validation of a nomogram for melanoma prognosis integrating risk score and clinicopathological factors

**Fig. 3:**
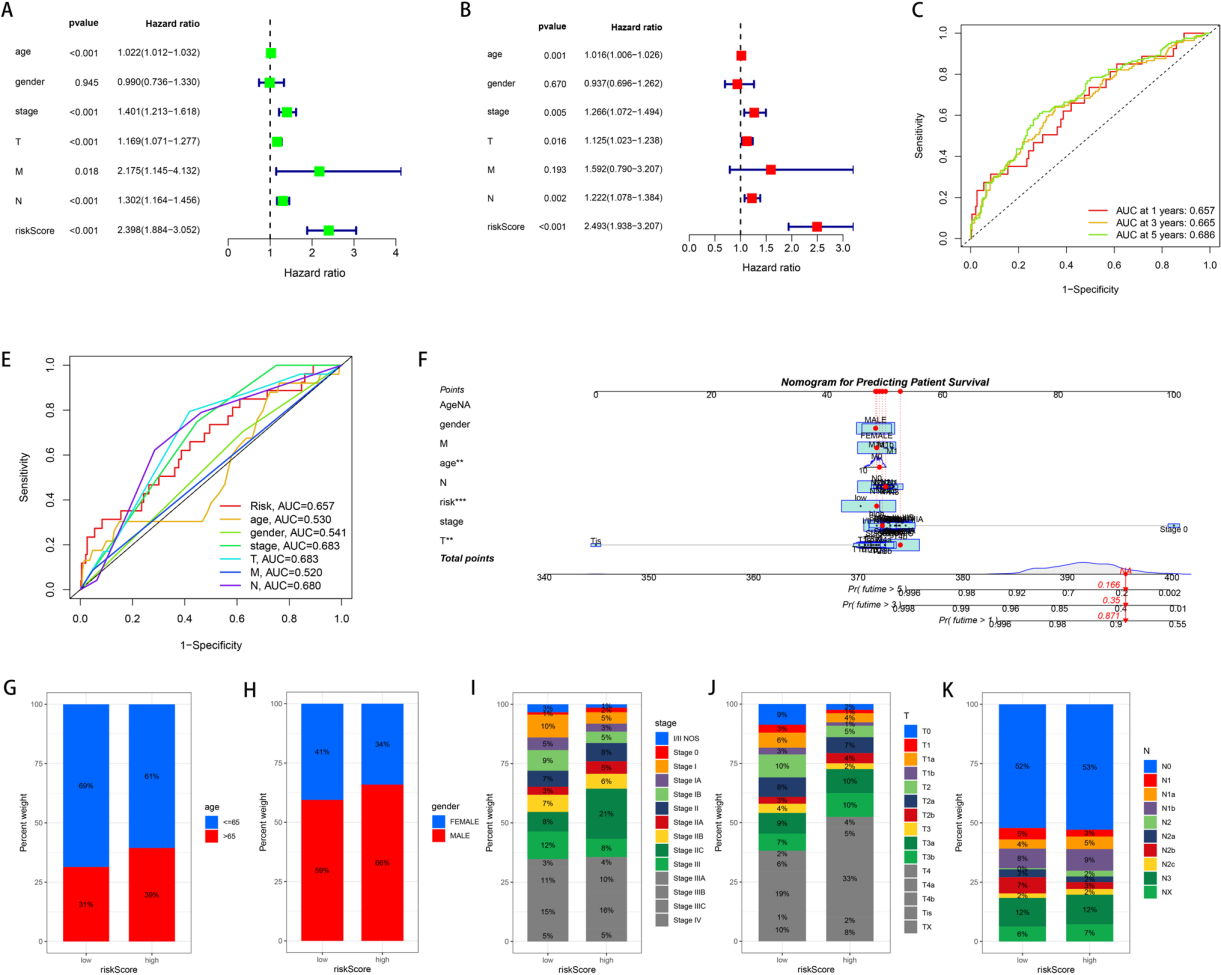
Prognostic validation of the risk model and its association with clinical features. **A** Forest plot of univariate Cox regression analysis for candidate genes. **B** Forest plot of multivariate Cox regression analysis showing independent prognostic genes incorporated into the model. **C** Time-dependent ROC curves evaluating the predictive performance of the risk model at 1-, 3-, and 5-year overall survival. **D**–**G** Bar plots illustrating the distribution of risk scores across clinicopathological subgroups, including age, gender, AJCC stage, and tumor subtype

Following our initial gene expression-based risk stratification, we conducted both univariate and multivariate Cox proportional hazards analyses to ascertain independent prognostic factors. As demonstrated in Fig. 3A, B, age, stage, T classification, N classification, and the derived risk score were identified as significant independent prognostic indicators. The validity of these factors was further supported by time-dependent receiver operating characteristic (ROC) curves, which displayed satisfactory predictive accuracy for 1-year, 3-year, and 5-year survival (Fig. 3C). To enhance clinical applicability, we established a nomogram that incorporates the risk score alongside age, gender, and clinicopathological staging (Fig. 3F). The clinical utility of the nomogram was evidenced by ROC curve analyses (Fig. 3E), indicating that the integration of the risk score with clinicopathological factors significantly improved prognostic predictions compared to the risk score alone. Subsequent analyses highlighted the association between the risk score and clinicopathological characteristics. Bar plots (Fig. 3G–K) reveal the proportion of patients stratified into high and low-risk categories across different age groups, genders, and TNM stages. These visualizations underscore the correlation between higher risk scores and advanced age, male gender, and more progressed tumor stages.

### Correlative analysis of immune cell infiltration in melanoma by risk stratification using multiple algorithmic approaches

**Fig. 4:**
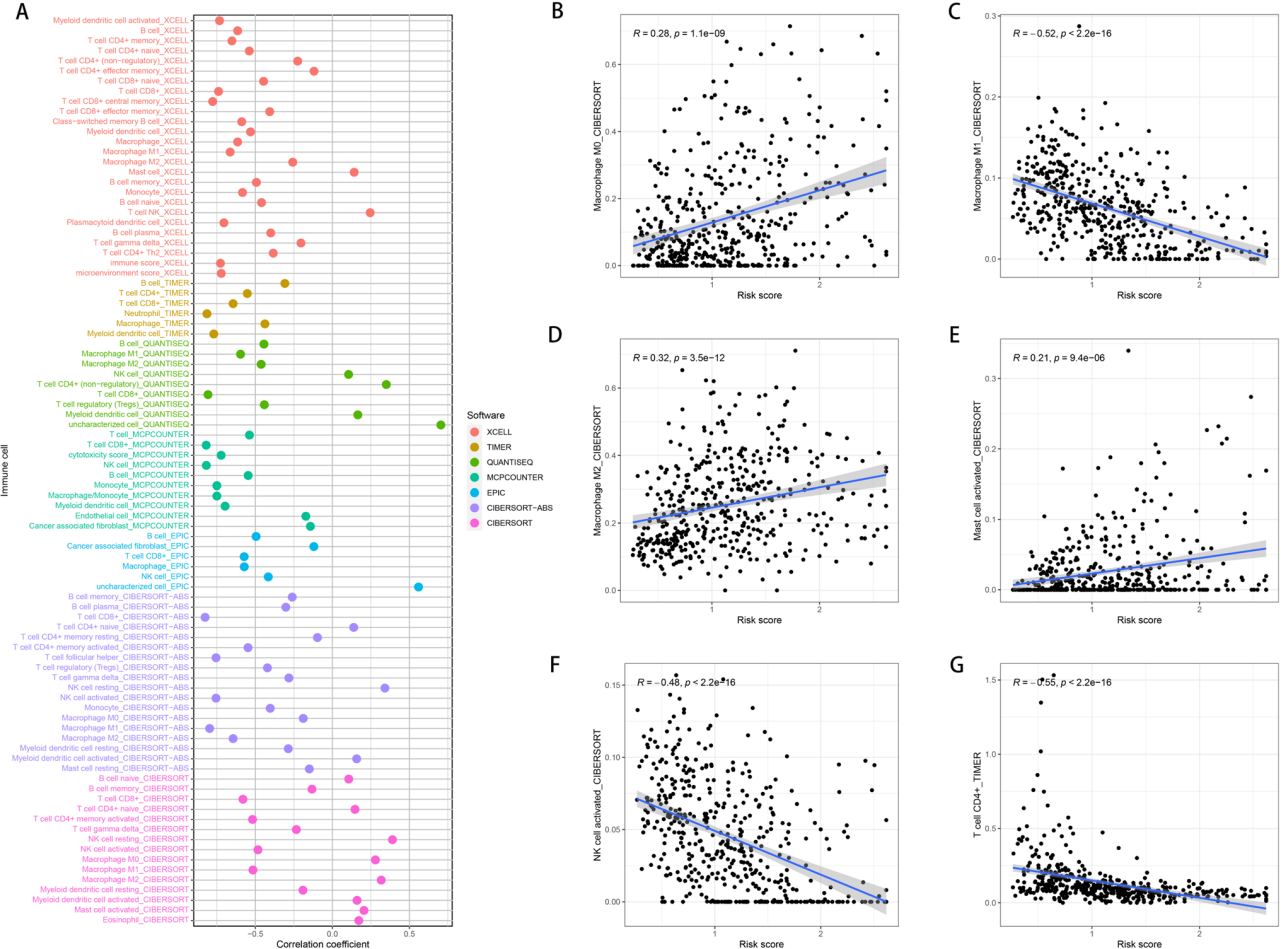
Correlation between immune cell infiltration and risk score. **A** Bubble plot showing the correlation between estimated immune cell populations and risk scores using multiple immune deconvolution algorithms (e.g., TIMER, CIBERSORT, xCell). **B**–**G** Scatter plots presenting the correlation between specific immune cell types (including CD8⁺ T cells, macrophages, and dendritic cells) and the calculated risk score. Spearman correlation coefficients and *p*-values are indicated

Expanding on our prognostic modeling, we conducted a comprehensive immune cell infiltration analysis utilizing a variety of algorithms to compare high and low-risk melanoma groups. Figure 4A presents a bubble plot illustrating the correlation of different immune cell types with the risk score. This plot encompasses data processed through multiple software platforms such as xCell, TIMER, QUANTISEQ, MCPCOUNTER, EPIC, CIBERSORT, and CIBERSORT-ABS, each contributing to a multi-dimensional view of the immune landscape. The correlation analysis revealed distinct patterns of immune cell prevalence between the two risk groups. For instance, myeloid-derived suppressor cells (MDSCs), as identified by xCell, showed a significant positive correlation with the risk score, suggesting their potential role in tumor progression in the high-risk group. Conversely, T cell CD8+ and T cell CD4+ memory resting populations, quantified by CIBERSORT, were negatively correlated with the risk score, indicating a possible reduction in immune surveillance within high-risk patients. Scatter plots (Fig. 4B–G) further delineate these relationships, with each panel representing a different immune cell type’s correlation with the risk score. Notably, the negative correlation of T cell CD8+ with the risk score (Fig. 4G) was consistent across multiple analysis platforms, underscoring its potential as a key biomarker for immune activity in melanoma.

### Gene set enrichment and variation analysis elucidates signaling pathways associated with risk stratification in melanoma

**Fig. 5:**
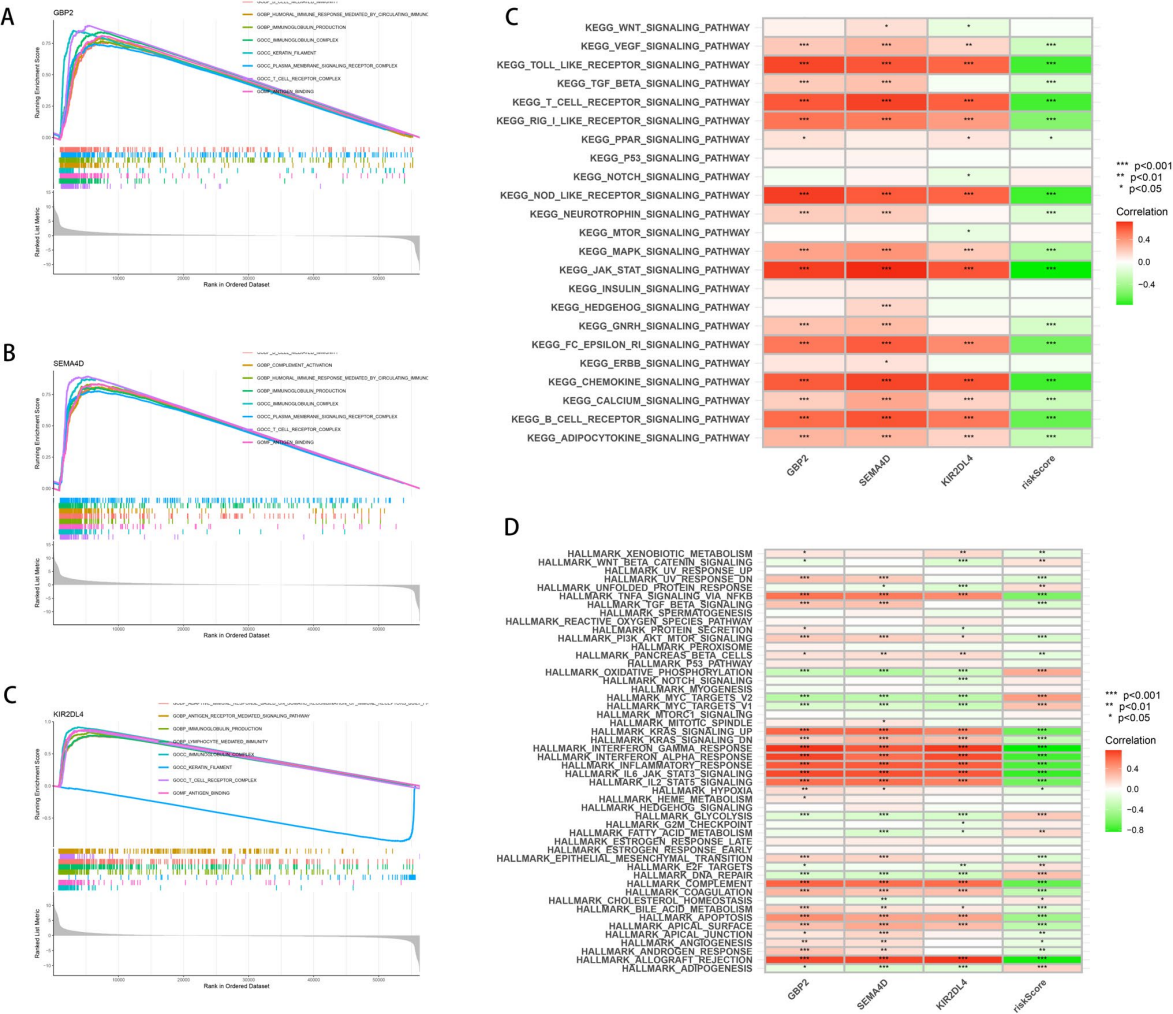
Pathway enrichment analysis associated with risk stratification. **A**–**C** GSEA enrichment plots of representative immune- and tumor-related pathways in high-risk versus low-risk groups. **D**, **E** Heatmaps displaying GSVA scores of selected pathways across samples, highlighting distinct pathway activity between risk subgroups

To further understand the biological implications of our risk stratification model, we performed Gene Set Enrichment Analysis (GSEA) and Gene Set Variation Analysis (GSVA) on genes associated with the risk model. GSEA results, represented in Fig. 5A–C, revealed significant enrichment of several key signaling pathways. In high-risk groups, there was a notable enrichment of oncogenic pathways, including KEGG_TGF_BETA_SIGNALLING_PATHWAY and KEGG_WNT_SIGNALING_PATHWAY, which are critically involved in tumor progression and metastasis. Conversely, pathways involved in immune activation, such as the KEGG_T_CELL_RECEPTOR_SIGNALING_PATHWAY and KEGG_B_CELL_RECEPTOR_SIGNALING_PATHWAY, were less enriched in high-risk groups, suggesting potential immune evasion mechanisms in more aggressive melanoma subtypes. The GSVA (Fig. 5D, E) corroborated these findings, with high-risk groups demonstrating activation of pathways associated with cancer proliferation and metastasis, such as HALLMARK_MTORC1_SIGNALING and HALLMARK_HYPOXIA.

### Prognostic impact of GBP2, SEMA4D, and KIR2DL4 expression in melanoma and their correlation with disease progression

**Fig. 6:**
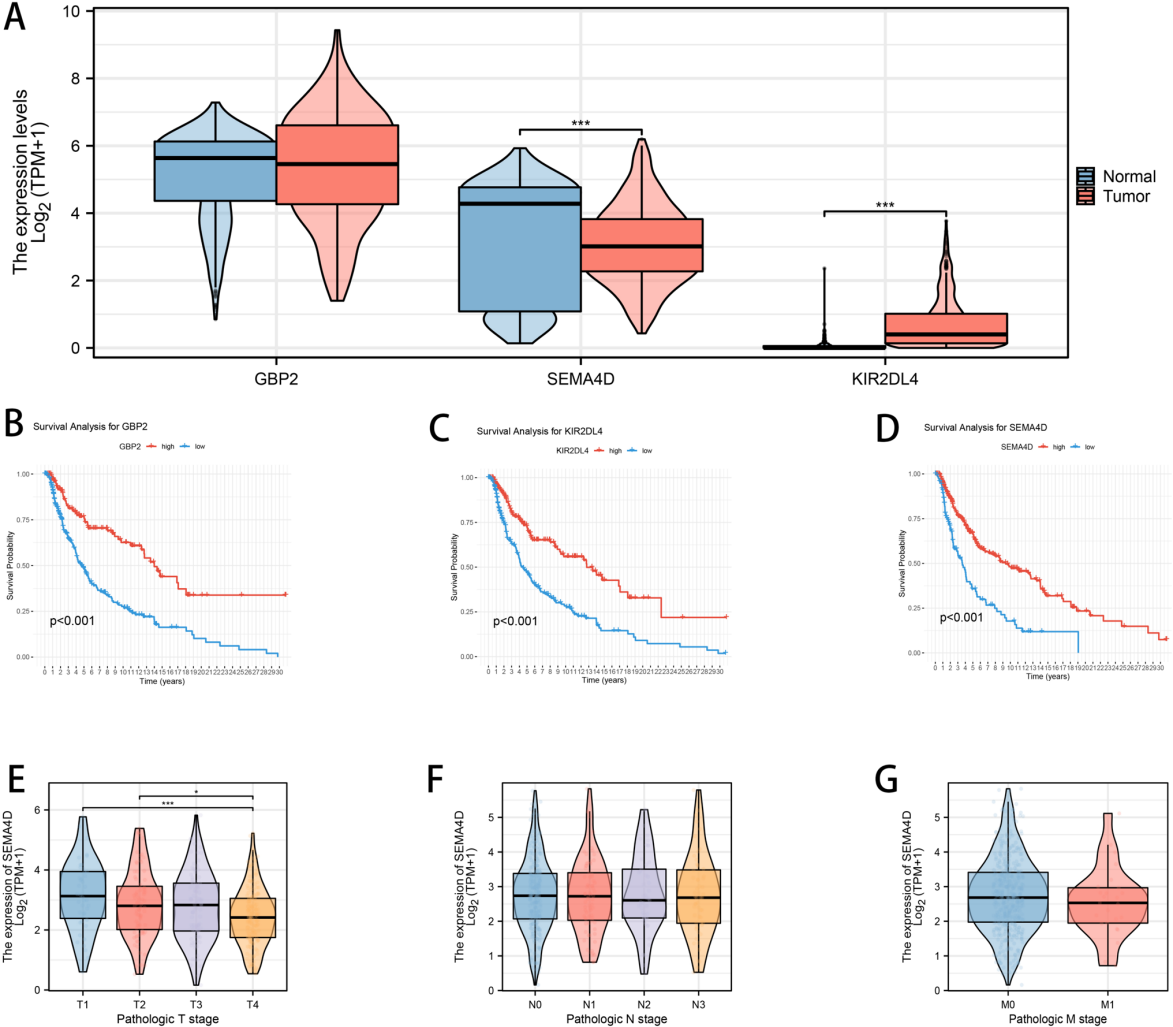
Gene expression and survival analysis of key prognostic biomarkers. **A** Violin plots comparing the expression levels of GBP2, SEMA4D, and KIR2DL4 in normal versus melanoma tissues. **B**–**D** Kaplan–Meier survival curves showing the association between high versus low expression of each gene and patient overall survival. **E**–**G** Violin plots illustrating the relationship between gene expression levels and clinical TNM staging (T, N, and M), revealing progressive upregulation with advanced pathological stages

An in-depth analysis of three genes from our risk model—GBP2, SEMA4D, and KIR2DL4—revealed distinct expression patterns and associations with melanoma prognosis. Figure 6A indicates that the expression levels of these genes were significantly higher in tumor samples compared to normal tissue, with SEMA4D and KIR2DL4 showing particularly pronounced elevation in tumor specimens, suggesting their involvement in tumorigenesis or tumor progression. Survival analyses for these genes (Fig. 6B–D) demonstrated that higher expression levels were associated with a significantly poorer prognosis in melanoma patients. This was most notable for KIR2DL4, where high expression correlated with a markedly decreased survival rate, implicating its potential role as a prognostic biomarker. Further, we examined the expression of these genes across different stages of melanoma. Expression levels of GBP2 and SEMA4D stratified by pathologic T stage (Fig. 6E) and N stage (Fig. 6F) showed a trend of increasing expression with advancing disease stage, although this trend was not statistically significant for all stages. KIR2DL4 expression also varied with pathologic M stage (Fig. 6G), providing additional evidence of its correlation with disease progression.

### KIR2DL4 knockdown suppresses proliferation and migration of melanoma cells

**Fig. 7:**
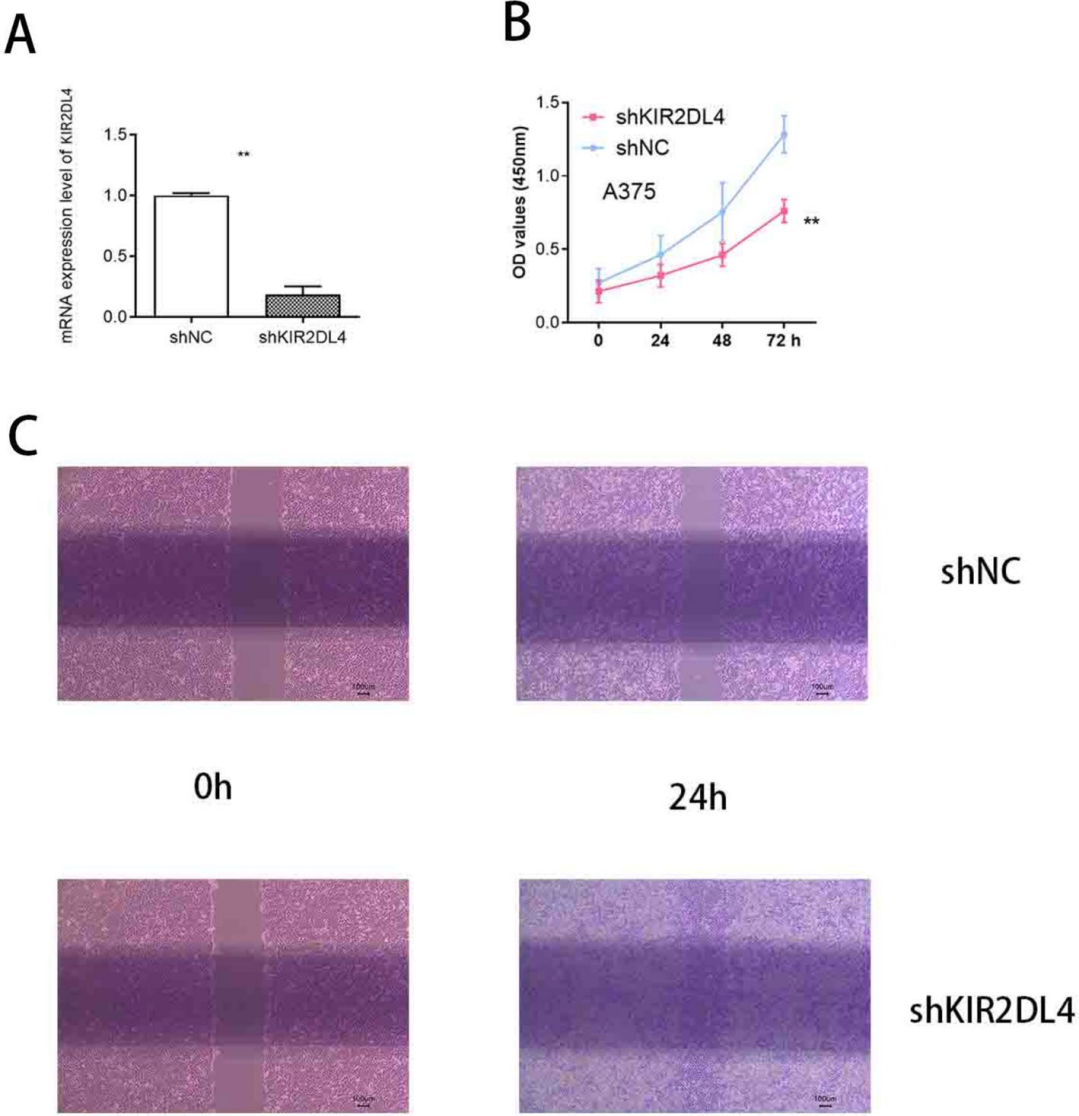
Knockdown of KIR2DL4 inhibits proliferation and migration of A375 melanoma cells. **A** Quantitative real-time PCR analysis confirmed the efficient knockdown of KIR2DL4 mRNA in A375 cells transduced with shKIR2DL4 compared to negative control (shNC). *p* < 0.01. **B** Cell proliferation was assessed using the MTT assay at 0, 24, 48, and 72 h post-transduction. Knockdown of KIR2DL4 significantly reduced the proliferative capacity of A375 cells compared to shNC. *p* < 0.01. **C** Wound healing assay showing cell migration at 0 and 24 h after scratching. The shKIR2DL4 group exhibited impaired wound closure compared to the shNC group, indicating reduced migratory ability

To investigate the functional role of KIR2DL4 in melanoma cells, we knocked down KIR2DL4 expression in A375 cells using a specific shRNA (shKIR2DL4), with a non-targeting shRNA (shNC) as the control. Quantitative real-time PCR confirmed that KIR2DL4 mRNA expression was significantly reduced in the shKIR2DL4 group compared to the control group (*p* < 0.01) (Fig. 7A). Next, we assessed the effect of KIR2DL4 knockdown on cell proliferation using the MTT assay. The results showed that silencing KIR2DL4 markedly inhibited the proliferative ability of A375 cells at 48 and 72 h post-transduction, compared with the shNC group (*p* < 0.01) (Fig. 7B). To further examine the impact of KIR2DL4 on cell migration, a wound healing assay was performed. After 24 h, the wound area in the shKIR2DL4 group remained significantly wider than that in the shNC group, indicating that KIR2DL4 knockdown impaired the migratory capacity of A375 cells (Fig. 7C).

## Discussion

In our study, we delved into the complex interplay between immune-related gene expression, immune cell infiltration, and signaling pathways in melanoma, aiming to enhance our understanding of the disease’s underlying biology and to improve prognostic accuracy.

The risk stratification model developed through this analysis has shed light on the molecular diversity of melanoma and its implications for patient survival. The differential expression of genes such as GBP2, SEMA4D, and KIR2DL4, which were found to be upregulated in tumor tissues, highlights their potential as prognostic biomarkers. The differential expression of genes such as GBP2, SEMA4D, and KIR2DL4, which were found to be upregulated in tumor tissues, highlights their potential as prognostic biomarkers [16–18].

Importantly, to complement bioinformatic predictions with experimental validation, we conducted functional assays focused on KIR2DL4, one of the key genes identified in our model. Using shRNA-mediated knockdown in A375 melanoma cells, we confirmed that KIR2DL4 promotes melanoma cell proliferation and migration, as demonstrated by reduced cell viability in MTT assays and impaired wound closure in wound healing experiments. These findings are consistent with previous reports suggesting a dual role of KIR2DL4 in modulating immune signaling and tumor progression [19, 20].

Their association with poorer survival outcomes reinforces the need for further investigation into their biological roles and therapeutic targeting potential. The correlation between gene expression and disease progression, particularly the increasing trend observed with advanced pathologic stages, underscores the aggressive nature of melanoma and the imperative for early detection and intervention. Moreover, our analysis has illuminated the significant impact of immune cell infiltration in melanoma.

The negative correlation of T cell CD8+ presence with the risk score, consistent across multiple algorithmic platforms, indicates a possible immunosuppressive tumor microenvironment in higher-risk patients. This finding aligns with the current understanding that a robust anti-tumor immune response, often marked by the presence of CD8+ T cells, is a favorable prognostic factor. Consequently, therapeutic strategies that enhance T cell infiltration and activity, such as immune checkpoint inhibitors, could be particularly effective. The integration of gene set enrichment analysis (GSEA) and gene set variation analysis (GSVA) has allowed us to pinpoint pathways that are differentially activated in high-risk melanoma.

The enrichment of oncogenic pathways such as TGF-beta and WNT signaling in high-risk groups points to their involvement in melanoma pathogenesis and suggests they could be promising targets for novel treatments. Finally, the construction and validation of a nomogram combining molecular and clinical data is a step towards personalized medicine in melanoma care. The nomogram’s ability to predict survival more accurately than the risk score alone is a testament to the power of combining diverse data types.

This approach facilitates a more nuanced risk assessment, enabling clinicians to tailor treatments to individual patients’ risk profiles. The discussion of these findings places them in the context of the broader scientific literature and emphasizes their potential to contribute to the development of precision oncology in melanoma. It also sets the stage for future research, which should aim to validate these biomarkers in prospective studies, explore the mechanistic roles of the identified genes, and assess the efficacy of targeted therapies that modulate the highlighted signaling pathways and immune responses.

## Limitation

Despite the strengths of this study, several limitations should be acknowledged. First, the prognostic model and immune stratification were developed primarily based on bulk RNA sequencing data from the TCGA cohort, which may introduce dataset-specific biases and limit generalizability. Although we performed external validation using the GEO dataset (GSE65904), prospective clinical validation in larger, real-world cohorts is necessary to confirm the robustness and clinical utility of the model. Second, our analysis focused on transcriptomic data, without incorporating proteomic, genomic, or epigenetic layers that could provide a more comprehensive understanding of melanoma heterogeneity and immune regulation. Third, while in vitro experiments were conducted to validate the tumor-promoting role of KIR2DL4, the functional roles of GBP2 and SEMA4D remain to be explored experimentally. Additionally, the tumor microenvironment is highly complex and dynamic, and bulk RNA data cannot capture cell-type-specific interactions or spatial heterogeneity. Single-cell and spatial transcriptomic technologies could further refine the understanding of immune landscape dynamics and biomarker relevance. Finally, the clinical application of the nomogram requires careful ethical consideration, especially in communicating high-risk classifications to patients and integrating molecular risk scores into routine decision-making.

## Data Availability

The data can be obtained from the correspondence author on request.
